# Development of a machine learning-based multicenter study for predicting severe intraventricular hemorrhage in preterm infants using perinatal variables

**DOI:** 10.3389/fneur.2026.1953566

**Published:** 2026-10-01

**Authors:** Fei Shen, Cheng Jin, Feixiang Wang, Rui Cheng, Hui Rong, Yang Yang, Caihong Fu

**Affiliations:** 1 Department of Neonatology, Children’s Hospital of Nanjing Medical University, Nanjing, Jiangsu, China; 2 Department of Urology, Children’s Hospital of Nanjing Medical University, Nanjing, Jiangsu, China; 3 Department of Hematology and Oncology, Children’s Hospital of Nanjing Medical University, Nanjing, Jiangsu, China

**Keywords:** machine learning, nomogram, prediction model, preterm infants, severe intraventricular hemorrhage

## Abstract

**Aim:**

To develop and validate machine learning models for predicting severe intraventricular hemorrhage (SIVH) in preterm infants using only prenatal and intrapartum variables, and to identify the optimal model.

**Method:**

This multicenter retrospective cohort study included preterm infants (gestational age ≤34 weeks) who required noninvasive or invasive respiratory support, using data from the Jiangsu Neonatal Respiratory Failure Collaborative Network (2019–2021). Data were split 7:3 into training and validation sets. Eight machine learning algorithms were compared. Performance was assessed by area under the curve (AUC), calibration, and decision curve analysis. An independent cohort served for temporal validation.

**Results:**

Among 3,320 infants, the incidence of SIVH was 6.66%. Six predictors were identified: gestational age, birth weight, endotracheal intubation in the delivery room, chest compressions in the delivery room, surfactant administration in the delivery room, and chorioamnionitis. In the training set, logistic regression achieved the highest mean AUC based on 10-fold cross-validation. Bootstrap internal validation of the LR model yielded a corrected AUC of 0.809. In the independent hold-out validation set, the LR model again demonstrated the best discriminative ability among the eight algorithms (AUC: 0.804), with satisfactory calibration and favorable net benefit. Sensitivity analyses showed that the LR model maintained robust performance in temporal validation using an independent cohort (AUC: 0.822) and across geographic subgroups (AUC range: 0.729–0.885). An online interactive tool was also developed to facilitate clinical application.

**Interpretation:**

A LR model using six readily available perinatal variables provides good discrimination and calibration for predicting SIVH in preterm infants, enabling immediate risk stratification at birth for SIVH occurring during the neonatal hospitalization period.

## Highlights

- First multicenter comparison of eight machine learning models for SIVH prediction.
- Six perinatal predictors enable immediate risk stratification at birth.
- Model shows robust performance across temporal and geographic subgroups.

## Introduction

Severe intraventricular hemorrhage (SIVH) is a major contributor to mortality and long-term neurodevelopmental impairments in preterm infants (1–2). Despite continuous advancements in neonatal intensive care, the incidence of SIVH remains high among preterm infants and increases with decreasing gestational age. Worldwide, the incidence of SIVH in preterm infants ≤28 weeks of gestation ranges from 5 to 52% (3). SIVH typically occurs within the first 72 h after birth and lacks specific clinical manifestations in the early stage, making it detectable only through routine cranial ultrasound screening. Clinical guidelines recommend the first imaging screening for preterm infants <32 weeks of gestation between 4 to 7 days after birth. This strategy represents a post-hemorrhage diagnosis, failing to achieve precise pre-hemorrhage warning and thus missing the critical window for hemorrhage prevention (4). Therefore, there is an urgent need to develop predictive tools that enable accurate risk stratification for SIVH immediately at birth.

In recent years, multiple studies have focused on exploring the risk factors for SIVH and developing prediction models. Existing evidence indicates that lower gestational age and birth weight, inadequate antenatal corticosteroid use, low Apgar scores, invasive mechanical ventilation, electrolyte disturbances, thrombocytopenia, and perinatal infection are high-risk factors for SIVH (5–7). Based on these factors, several models have shown preliminary predictive potential, but current studies still have notable limitations. Our recent systematic review demonstrated that all existing studies on prediction models for SIVH in preterm infants are at high risk of bias, with improper handling of missing data, use of univariate analysis for predictor selection, inadequate calibration assessment, and lack of robust internal validation methods being the main sources of bias (8). A systematic review by Xiong et al. similarly evaluated previously developed prediction models for intraventricular hemorrhage (IVH) in preterm infants and concluded that existing models have certain shortcomings (9). They recommended that future studies increase sample sizes and the number of outcome events, properly handle missing data, and optimize statistical analyses to facilitate model generalizability and clinical application (9). Furthermore, the vast majority of previous models have incorporated postnatal variables such as invasive ventilation parameters, hematological indicators, and the use of inotropic agents. These variables have complex temporal causal relationships with the occurrence of SIVH, and their acquisition itself involves time delays, limiting the early warning capability of the models during the critical golden window after birth.

This study aims to develop and validate a prediction model for SIVH in preterm infants requiring respiratory support, using only prenatal and intrapartum variables. To systematically compare the predictive performance of different algorithms for SIVH and select the optimal model, we will employ eight commonly used machine learning (ML) methods for model development. The results of this study will provide clinicians with an early, noninvasive risk assessment tool for SIVH, applied at birth to stratify infants according to their risk of hemorrhage during the neonatal period. This may assist in individualizing preventive strategies and monitoring approaches.

## Materials and methods

### Study design and setting

This study is a multicenter retrospective cohort study aimed at developing and validating a model for predicting the risk of SIVH in preterm infants immediately after birth. The data were sourced from the Jiangsu Neonatal Respiratory Failure Collaborative Network (JSNRFCN; registration number: 13622464; date of registration: December 9, 2022). Hospitals participating in the JSNRFCN must be tertiary referral hospitals with comprehensive neonatal diagnostic and treatment capabilities and recognized expertise in high-risk neonatal care. In 2019, 30 neonatal intensive care units (NICUs) from all 13 cities in Jiangsu Province joined this collaborative network. The network continuously collects standardized data on demographic characteristics, clinical outcomes, specific clinical practices, and resource utilization for infants born at participating hospitals with gestational ages ranging from 23 weeks + 5 days to 41 weeks + 6 days who receive respiratory support (noninvasive or invasive). This study was approved by the Ethics Committee of Children’s Hospital of Nanjing Medical University (Number: 202004037–1). Although informed consent was waived due to the retrospective observational nature of the study, ethical standards were upheld. The study report adheres to the Transparent Reporting of a Multivariable Prediction Model for Individual Prognosis or Diagnosis (TRIPOD) statement (10).

### Study participants and sample size

This study included all preterm infant records registered in the JSNRFCN between January 1, 2019, and December 31, 2021. As per the network’s enrollment criteria, all registered infants received noninvasive or invasive respiratory support. Preterm infants meeting all of the following criteria were included in the study: (1) gestational age at birth ≤34 weeks; (2) age at admission ≤24 h. Infants meeting any of the following criteria were excluded: (1) presence of severe congenital malformations or inherited metabolic diseases; (2) no cranial ultrasound examination performed or no cranial ultrasound results recorded during hospitalization. Additionally, an independent cohort of patients admitted to the Children’s Hospital of Nanjing Medical University between January 1, 2022 and December 31, 2023, meeting the aforementioned inclusion and exclusion criteria, was included for temporal validation.

The sample size was calculated using R software (version 4.4.2) according to the formula provided in the BMJ clinical prediction model guideline (11). Since we included preterm infants within the same gestational age range as Singh et al. (12), we referenced their SIVH incidence of 5.59% for sample size calculation, i.e., the assumed outcome prevalence per predictor parameter was 0.0559. Assuming the model achieves a C-statistic of 0.8, an intercept estimation error of 0.05, and 5–10 predictor factors, the minimum required sample size for developing a new model is approximately 593–1,186 cases. This study adopted the higher value of 1,186 cases. Considering missing data and case exclusions (estimated at 10%), at least 1,318 subjects needed to be included.

### Variables

Based on guidelines and a systematic literature review, all available variables were extracted from the JSNRFCN database, primarily including antenatal treatment factors, maternal factors of preterm infants, intrapartum factors, and clinical outcomes of preterm infants. Antenatal factors mainly consisted of antenatal corticosteroid therapy. Maternal factors of preterm infants mainly included: maternal age, *in vitro* fertilization, presence of conditions such as hypertension, heart disease, severe anemia, gestational diabetes mellitus, clinical chorioamnionitis, mode of delivery, appearance and volume of amniotic fluid, and presence of multiple gestation, fetal distress, or placental abruption. Intrapartum factors mainly included: sex of the preterm infant, gestational age at birth and birth weight, 1-min and 5-min Apgar scores, delivery room (DR) resuscitation status, and administration of surfactant in the DR.

After data extraction for this study was completed, a second researcher performed random sampling verification to ensure the accuracy of data extraction.

Clinical chorioamnionitis was diagnosed when the pregnant woman had fever (body temperature >38 °C) accompanied by two or more of the following: maternal tachycardia (>100 beats/min), maternal leukocytosis (white blood cell count >15,000/mm^3^), purulent or foul-smelling amniotic fluid or vaginal discharge, uterine tenderness, or fetal tachycardia (>160 beats/min) (13).

### Delivery room resuscitation

Resuscitation of all preterm infants after birth was performed in accordance with the Chinese Neonatal Resuscitation Guidelines (14). After birth, positive pressure ventilation was administered to preterm infants via a face mask. The resuscitation gas was uniformly warmed and humidified before use, and subsequent respiratory support was provided via a T-piece. The decision of whether further endotracheal intubation was required was made by the neonatologist in the DR (14). Intubation was considered necessary if positive pressure ventilation was ineffective, prolonged T-piece positive pressure ventilation was required, or chest compressions were performed. After resuscitation, the infants were transferred to the NICUs for further evaluation by a neonatologist.

### Study outcome

The primary outcome of this study was the occurrence of SIVH within the first 7 days after birth. According to the criteria of Papile (15) as revised by Volpe (16), IVH can be classified into four grades. Grade I (hemorrhage confined to the germinal matrix) and Grade II (blood occupying ≤50% of the lateral ventricular volume) are considered mild, while Grade III (blood occupying >50% of the lateral ventricular volume) and Grade IV (hemorrhagic infarction occurring in the periventricular area ipsilateral to the hemorrhage) are considered severe. Hemorrhage of different grades can occur unilaterally or bilaterally. In this study, all cranial ultrasound examinations were performed within 1 week after birth by specialized ultrasonographers with at least 2 years of experience in neonatal neurosonography, using standardized bedside ultrasound equipment (GE or Philips, with high-frequency 5–7.5 MHz sector probes) and a uniform protocol including coronal and sagittal views through the anterior fontanelle. If signs of IVH were detected, repeat examinations were conducted every 2 days until the hemorrhage stabilized or resolved. The highest grade of hemorrhage observed during the 7-day period was recorded as the final diagnosis. All centers uniformly applied the Papile classification, and each center had at least two experienced ultrasonographers who resolved diagnostic disagreements by consensus; annual network meetings were held to harmonize interpretation standards across centers.

### Data preprocessing and missing data handling

All data cleaning and preprocessing steps were performed consistently across the development and validation sets, as detailed below: (1) Duplicate data processing: Duplicate entries were merged based on the unique infant identifier (hospital admission number), and the most recent complete record was retained. (2) Outlier handling: Boxplot methods combined with medically theoretically logical thresholds were used to identify extreme values and contradictory data. Invalid records were excluded after review by clinical experts. (3) Missing data handling: The missing data mechanism in this study was assumed to be missing at random (MAR). All predictors were included as imputation covariates. To prevent information leakage, multiple imputation by chained equations (MICE, *n* = 5) was performed after the train-validation split, and exclusively on the training set. The imputation model fitted on the training set was subsequently applied to impute missing values in the validation set. Based on the theoretical framework proposed by Noghrehchi et al. (17), information criteria (IC) were used to guide the selection of the multiple imputation dataset.

### Data analysis

Data organization and analysis were performed using R 4.4.2. Continuous variables following a normal distribution were expressed as mean ± standard deviation (SD), and comparisons between two groups were made using the independent samples *t*-test. Continuous variables not following a normal distribution were expressed as median and interquartile range (IQR), and comparisons between two groups were made using the Mann–Whitney U test. Categorical variables were expressed as n (%), and comparisons between groups were made using the chi-square test or Fisher’s exact test. A *p*-value <0.05 was considered statistically significant.

The missing data mechanism was assessed using Little’s Missing Completely at Random (MCAR) test. Given that missingness was plausibly related to observed clinical characteristics (e.g., gestational age and illness severity), we assumed a MAR mechanism and used multiple imputation accordingly. However, we acknowledge that the MAR assumption cannot be empirically verified and should be interpreted with caution. To assess the robustness of our findings to plausible departures from the MAR assumption, we performed a sensitivity analysis using NARFCS (Not At Random Fully Conditional Specification). A range of δ adjustment parameters was applied (δ = −1.5, −1.0, −0.5, 0, +0.5, +1.0, +1.5), assuming that missing values differed systematically from observed values by 0.5, 1.0, and 1.5 standard deviations. The model was refitted under each scenario, and the resulting estimates were compared with those obtained under MAR-based imputation.

The modeling dataset was divided into a training set and a validation set at a ratio of 7:3 using random stratified sampling. Model development was performed in the 70% development set. As a sensitivity analysis for class imbalance, synthetic minority over-sampling technique (SMOTE) (k = 5, duplication factor = 10) was applied only within cross-validation training folds, while the validation set remained unsampled to preserve the natural outcome distribution and avoid data leakage. Feature variable selection was conducted using the least absolute shrinkage and selection operator (LASSO). LASSO regression compresses variable coefficients by constructing a penalty function, shrinking coefficients of irrelevant variables to zero, thereby achieving variable selection and dimensionality reduction. Ten-fold cross-validation was used to select the optimal penalty parameter λ. Variables with non-zero coefficients retained after LASSO screening were used as independent variables, and multicollinearity among the included variables was tested using multiple regression models, quantified by the variance inflation factor (VIF). A VIF ≥ 10 indicated significant multicollinearity among variables, whereas a VIF < 10 indicated no significant multicollinearity. The selected variables were then sequentially incorporated into eight ML algorithms: logistic regression (LR), neural network (NN), gradient boosting machine (GBM), extreme gradient boosting (XGBoost), random forest (RF), support vector machine (SVM), K-nearest neighbor (KNN), and adaptive boosting (Adaboost). Hyperparameter optimization was performed using grid search combined with 10-fold cross-validation within the training set. The parameter combination yielding the highest cross-validated AUC was selected as the optimal configuration for each algorithm. An interactive web application was constructed using the “DynNom” package and deployed to the shinyapps.io cloud service platform.

Model performance evaluation was performed in the 30% hold-out validation set. Temporal validation was conducted using an independent cohort from 2022 to 2023. Geographic subgroup sensitivity analyses were performed by stratifying the development cohort into three regions: Southern Jiangsu, Central Jiangsu, and Northern Jiangsu. Bootstrap resampling (1,000 times) was used for internal validation of the training set to correct for optimism. Discrimination was assessed using the area under the curve (AUC) of the receiver operating characteristic (ROC) curve. Calibration was evaluated using the Hosmer-Lemeshow test (*p* > 0.05 indicated good calibration) along with a calibration curve, and the Brier score was calculated. Decision curve analysis (DCA) was used to evaluate the net benefit of the model at different threshold probabilities. After excluding overfitted models using the DeLong test, the optimal model was selected based on the principle of “highest discrimination and best calibration” in the validation set.

## Results

### Baseline characteristics

Between January 2019 and December 2021, a total of 3,857 preterm infants with gestational age ≤34 weeks and age at admission ≤24 h were admitted to the JSNRFCN. After screening based on inclusion and exclusion criteria, 3,320 infants were finally included in this study. The screening process is shown in Figure 1. The result of Little’s MCAR test (χ^2^ = 728, df = 394, *p* < 0.001) rejected the MCAR assumption, and the proportion of missing data for all variables was less than 15%. Missing data were distributed across 6 variables, as detailed in Supplementary Table 1. A total of 5 imputations were performed on the original data, with little difference in indicators across all imputation models. Among these, the first imputation dataset had the smallest akaike information criterion (AIC) and bayesian information criterion (BIC) values, performing best among all models, indicating better data fit and more reasonable complexity control. Therefore, the first imputation dataset was selected for model development in this study. See Supplementary Table 2 for details.

**Figure 1:**
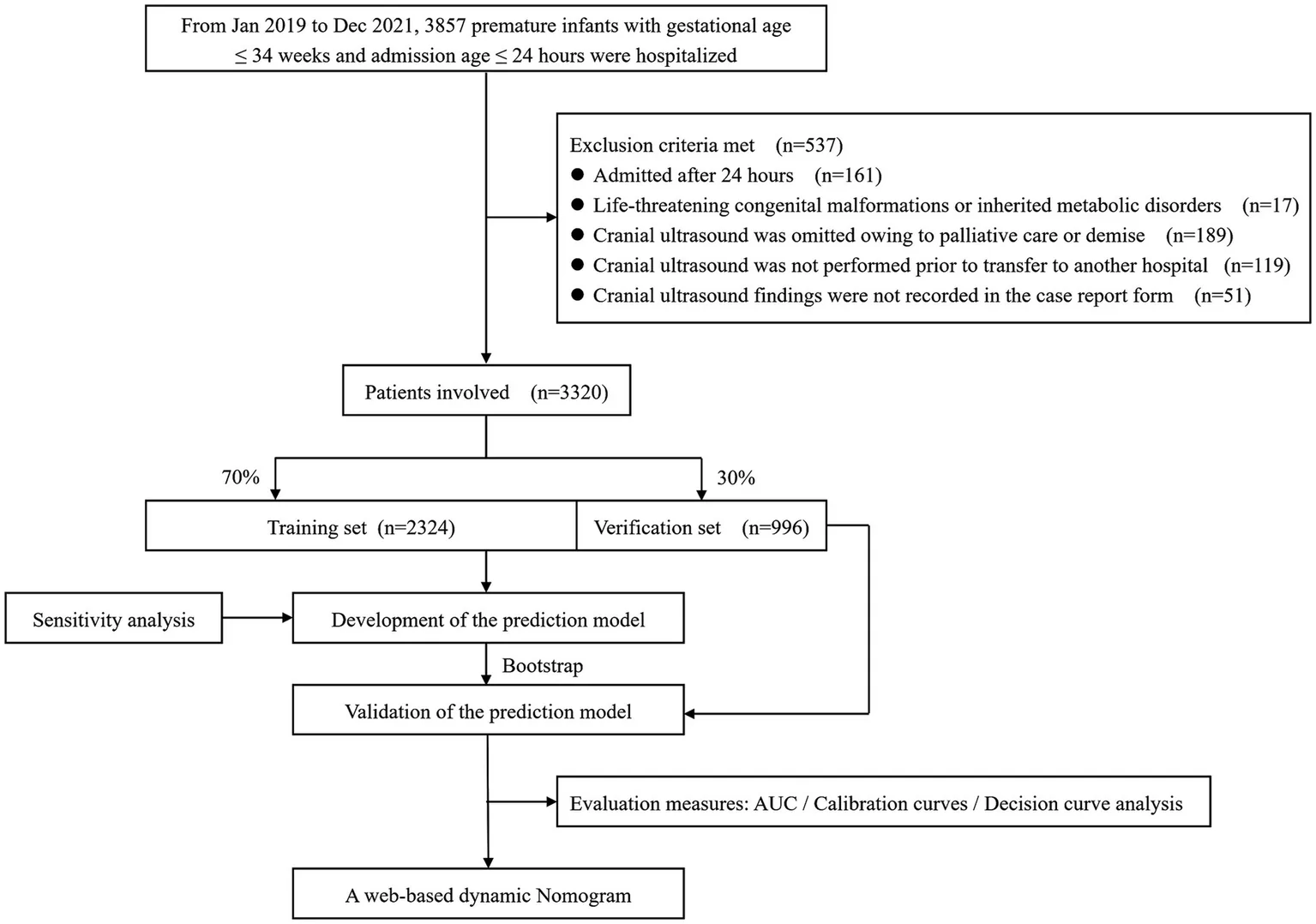
Flowchart of the study population selection.

Baseline characteristics of preterm infants and their mothers are shown in Table 1. In the overall cohort, the median gestational age at birth was 31.86 (30.14, 33.43) weeks, and the median birth weight was 1,650 (1,350, 2030) g. Among them, 221 cases (6.66%) developed SIVH, and 1,395 cases (42.02%) were female. The number of SIVH cases by gestational age stratum is shown in Supplementary Table 3. Detailed comparison of the characteristics of preterm infants between the training and validation sets is presented in Supplementary Table 4. In the training and validation sets, all baseline characteristics were balanced and comparable. To evaluate potential selection bias, we compared baseline characteristics between included (*n* = 3,320) and excluded infants (*n* = 359) using the raw, unimputed data for both groups. Among the excluded infants, 189 did not undergo cranial ultrasound because of palliative care or death, 119 were transferred to other hospitals before ultrasound examination could be performed, and 51 had ultrasound results that were unavailable for review. The comparison is presented in Supplementary Table 5. Excluded infants had significantly lower gestational age (median 29.71 vs. 31.86 weeks, *p* < 0.001) and birth weight (1,250 vs. 1,650 g, *p* < 0.001), and higher rates of DR intubation (22.0% vs. 6.3%, *p* < 0.001) and chest compressions (11.7% vs. 3.1%, *p* < 0.001), indicating that the excluded group represented a more critically ill subpopulation.

**Table 1:** Baseline characteristics of preterm infants and their mothers.

Variables	Total (*n* = 3,320)	Non-SIVH (*n* = 3,099)	SIVH (*n* = 221)	Statistic	*P*
Sex, *n* (%)				2.136	0.144
Female	1,395 (42)	1,313 (42)	82 (37)		
Male	1925 (58)	1786 (58)	139 (63)		
Gestational age, Median (Q1, Q3)	31.86 (30.14, 33.43)	31.86 (30.29, 33.43)	29.43 (27.57, 31)	533206.5	< 0.001
Multiple births, *n* (%)				0.03	0.863
No	2,547 (77)	2,379 (77)	168 (76)		
Yes	773 (23)	720 (23)	53 (24)		
Birth weight, Median (Q1, Q3)	1,650 (1,350, 2030)	1,690 (1,400, 2050)	1,230 (970, 1,550)	522056.5	< 0.001
SGA, *n* (%)				1.789	0.181
No	3,099 (93)	2,898 (94)	201 (91)		
Yes	221 (7)	201 (6)	20 (9)		
IVF, *n* (%)				6.948	0.008
No	2,837 (85)	2,662 (86)	175 (79)		
Yes	483 (15)	437 (14)	46 (21)		
Delivery mode, *n* (%)				16.281	< 0.001
Vaginal delivery	1,150 (35)	1,046 (34)	104 (47)		
Elective cesarean section	1,332 (40)	1,258 (41)	74 (33)		
Emergency cesarean section	838 (25)	795 (26)	43 (19)		
Fetal distress, *n* (%)				0.208	0.648
No	3,025 (91)	2,826 (91)	199 (90)		
Yes	295 (9)	273 (9)	22 (10)		
Clear amniotic fluid, *n* (%)				2.139	0.144
No	320 (10)	292 (9)	28 (13)		
Yes	3,000 (90)	2,807 (91)	193 (87)		
Normal amniotic fluid volume, *n* (%)				0	1
No	261 (8)	244 (8)	17 (8)		
Yes	3,059 (92)	2,855 (92)	204 (92)		
1 min Apgar, Median (Q1, Q3)	8 (7, 9)	8 (7, 9)	7 (6, 8)	432214.5	< 0.001
5 min Apgar, Median (Q1, Q3)	9 (8, 9)	9 (8, 9)	8 (8, 9)	425,200	< 0.001
No Resuscitation, *n* (%)				22.844	< 0.001
No	1,449 (44)	1,318 (43)	131 (59)		
Yes	1871 (56)	1781 (57)	90 (41)		
Endotracheal intubation in the DR, *n* (%)				63.128	< 0.001
No	3,112 (94)	2,933 (95)	179 (81)		
Yes	208 (6)	166 (5)	42 (19)		
Chest compressions in the DR, *n* (%)				39.463	< 0.001
No	3,217 (97)	3,019 (97)	198 (90)		
Yes	103 (3)	80 (3)	23 (10)		
PS administration in the DR, *n* (%)				Fisher	0.005
No	3,295 (99)	3,080 (99)	215 (97)		
Yes	25 (1)	19 (1)	6 (3)		
Antenatal steroids, *n* (%)				1.222	0.543
No	806 (24)	756 (24)	50 (23)		
Incomplete	1,004 (30)	930 (30)	74 (33)		
Complete	1,510 (45)	1,413 (46)	97 (44)		
Mother age, Median (Q1, Q3)	30 (27, 33)	30 (27, 33)	30 (27, 34)	326,385	0.242
GDM, *n* (%)				0.012	0.914
No	2,763 (83)	2,578 (83)	185 (84)		
Yes	557 (17)	521 (17)	36 (16)		
Placental abruption, *n* (%)				0.07	0.791
No	3,174 (96)	2,964 (96)	210 (95)		
Yes	146 (4)	135 (4)	11 (5)		
Gestationa hypertension, *n* (%)				1.704	0.192
No	2,799 (84)	2,620 (85)	179 (81)		
Yes	521 (16)	479 (15)	42 (19)		
Chronic hypertension, *n* (%)				Fisher	0.797
No	3,258 (98)	3,040 (98)	218 (99)		
Yes	62 (2)	59 (2)	3 (1)		
Maternal heart diseases,*n* (%)				Fisher	0.364
No	3,301 (99)	3,082 (99)	219 (99)		
Yes	19 (1)	17 (1)	2 (1)		
Maternal severe anemia, *n* (%)				Fisher	0.173
No	3,283 (99)	3,062 (99)	221 (100)		
Yes	37 (1)	37 (1)	0 (0)		
Chorioamnionitis, *n* (%)				38.607	< 0.001
No	3,154 (95)	2,964 (96)	190 (86)		
Yes	166 (5)	135 (4)	31 (14)		

IVF, in vitro fertilization; SGA, small for gestational age; PS, pulmonary surfactant; SIVH, severe intraventricular hemorrhage; GDM, gestational diabetes mellitus; DR, delivery room.

According to the same criteria, the independent cohort from January 1, 2022 to December 31, 2023 included a total of 233 preterm infants with gestational age ≤34 weeks, and their baseline characteristics are shown in Supplementary Table 6.

The distribution of infrequent DR exposures (surfactant administration and chest compressions) across the training and validation sets is presented in Supplementary Table 7. In the overall cohort, 25 infants (0.75%) received surfactant in the DR, of whom 6 (24.0%) developed SIVH; 103 infants (3.10%) received chest compressions, of whom 23 (22.3%) developed SIVH.

### Predictor screening

LASSO regression with L1 regularization constraint (λmin = 0.007745186) identified six non-zero coefficient variables, including gestational age at birth, birth weight, endotracheal intubation in the DR, chest compressions in the DR, PS administration in the DR, and Chorioamnionitis, as shown in Figure 2. The VIF values for all six predictors were <5, indicating no significant multicollinearity issues.

**Figure 2:**
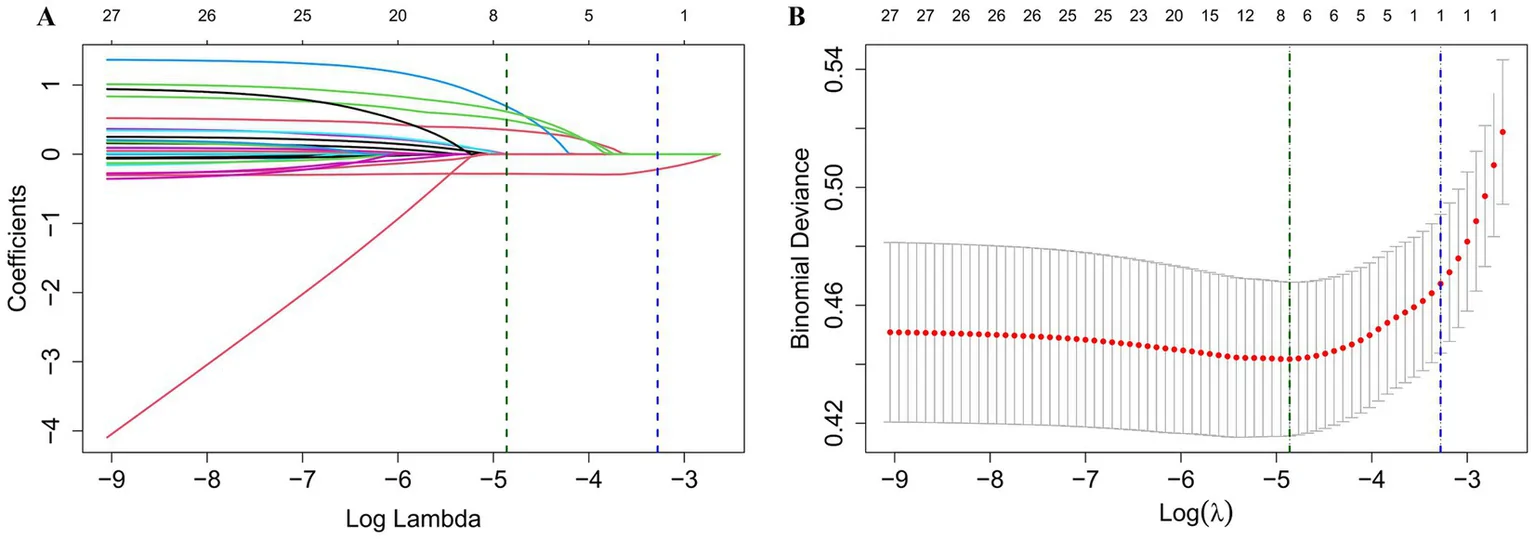
LASSO regression for variable selection. **(A)** Ten-fold cross-validation for λ selection; **(B)** LASSO coefficient path plot.

### Performance evaluation of the prediction models

Hyperparameter optimization for the eight models was performed on the training set using the six aforementioned predictors. The complete search grids and final parameter settings are provided in Supplementary Tables 8, 9, with cross-validation performance metrics summarized in Supplementary Table 10. In brief, LR achieved the highest training-set mean AUC (0.785) with default parameters. In the validation set, LR again demonstrated the best discriminative ability, with an AUC of 0.804 (95% CI: 0.738–0.869) (Figure 3A). To comprehensively evaluate predictive performance across the eight ML models in the validation cohort, we additionally calculated the F1 score, precision, specificity, sensitivity, and accuracy, as presented in Supplementary Figure 1. With respect to calibration, the LR model likewise showed superior performance (Supplementary Figure 2A). DCA for the eight models are displayed in Supplementary Figure 3A, where the LR model again demonstrated the most favorable net clinical benefit.

**Figure 3:**
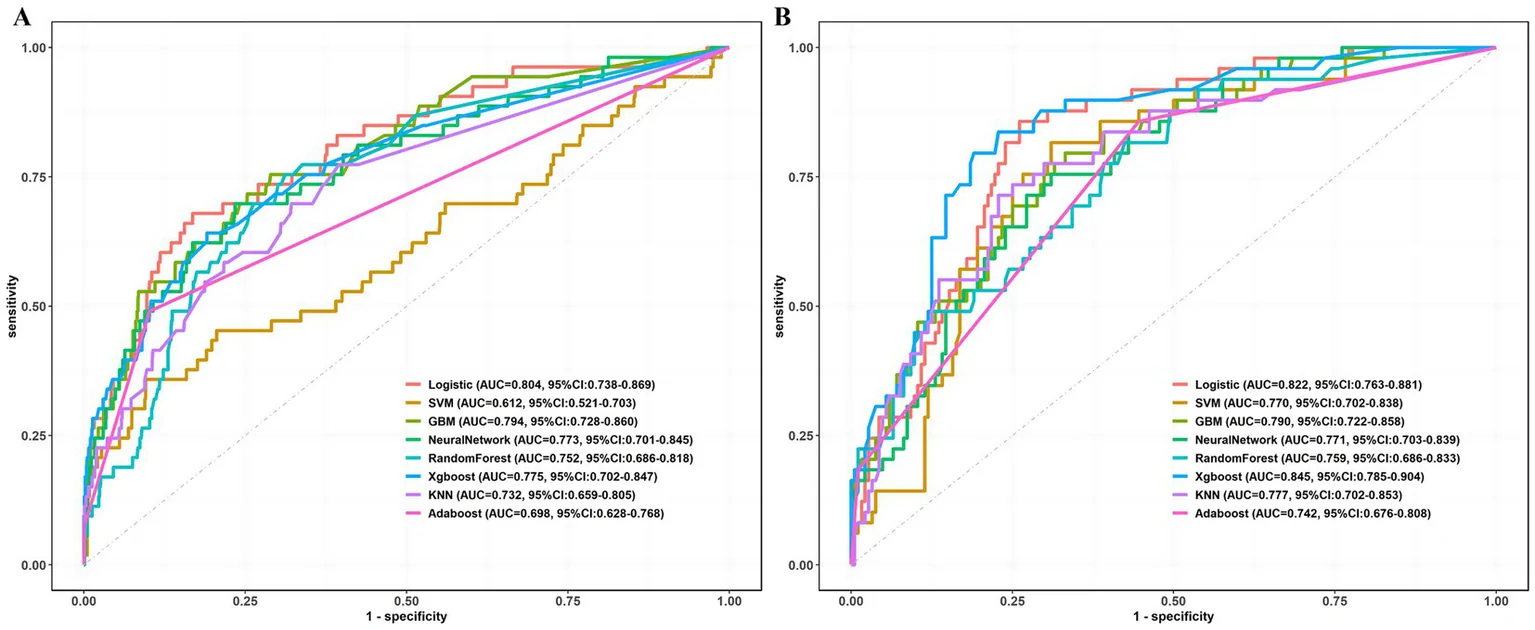
ROC curves of the eight machine learning models. **(A)** Internal validation set; **(B)** Temporal validation cohort.

Temporal validation using the independent cohort yielded an AUC of 0.822 (95% CI: 0.763–0.881) for the LR model, which was slightly lower than that of the XGBoost model (AUC: 0.845, 95% CI: 0.785–0.904) but superior to all other algorithms (Figure 3B). Furthermore, the LR model ranked among the top performers across all eight ML models in terms of F1 score, precision, specificity, sensitivity, and accuracy, as presented in Supplementary Figure 4. The calibration curves and DCA curves for the eight models are shown in Supplementary Figures 2B, 3B, both indicating favorable calibration and net clinical benefit for the LR model. Specifically, the LR model achieved a Brier score of 0.132, a calibration intercept of 0.071, a calibration slope of 1.088, and a Hosmer-Lemeshow test *p*-value of 0.133. After excluding infants with mild IVH from the temporal validation cohort, 49 infants remained, including 43 with grade III IVH and 6 with grade IV IVH. When grade III was used as the negative reference and grade IV as the positive outcome, the LR model achieved an AUC of 0.911 (95% CI: 0.772–1.000), a Brier score of 0.061, and a Hosmer-Lemeshow test *p*-value of 0.174. The corresponding ROC curve, calibration curve, and DCA curve are displayed in Supplementary Figure 5.

After calibration of the optimal LR model using 1,000 bootstrap resampling iterations for internal validation, the AUC value was 0.809 (95% CI: 0.776–0.843), as shown in Supplementary Figure 6. The calibration curves of the LR model in both the training and validation sets were close to the ideal line, as shown in Figure 4. The Brier score in the training set was 0.058, with a calibration intercept of 0.000 and a calibration slope of 1.000, and the Hosmer-Lemeshow test *p*-value was 0.439. In the validation set, the Brier score was 0.043, the calibration intercept was −0.031, the calibration slope was 1.103, and the Hosmer-Lemeshow test *p*-value was 0.423. The DCA result showed that when the threshold probability for predicting SIVH ranged from 10 to 70%, the prediction model provided net benefit, as shown in Figure 5.

**Figure 4:**
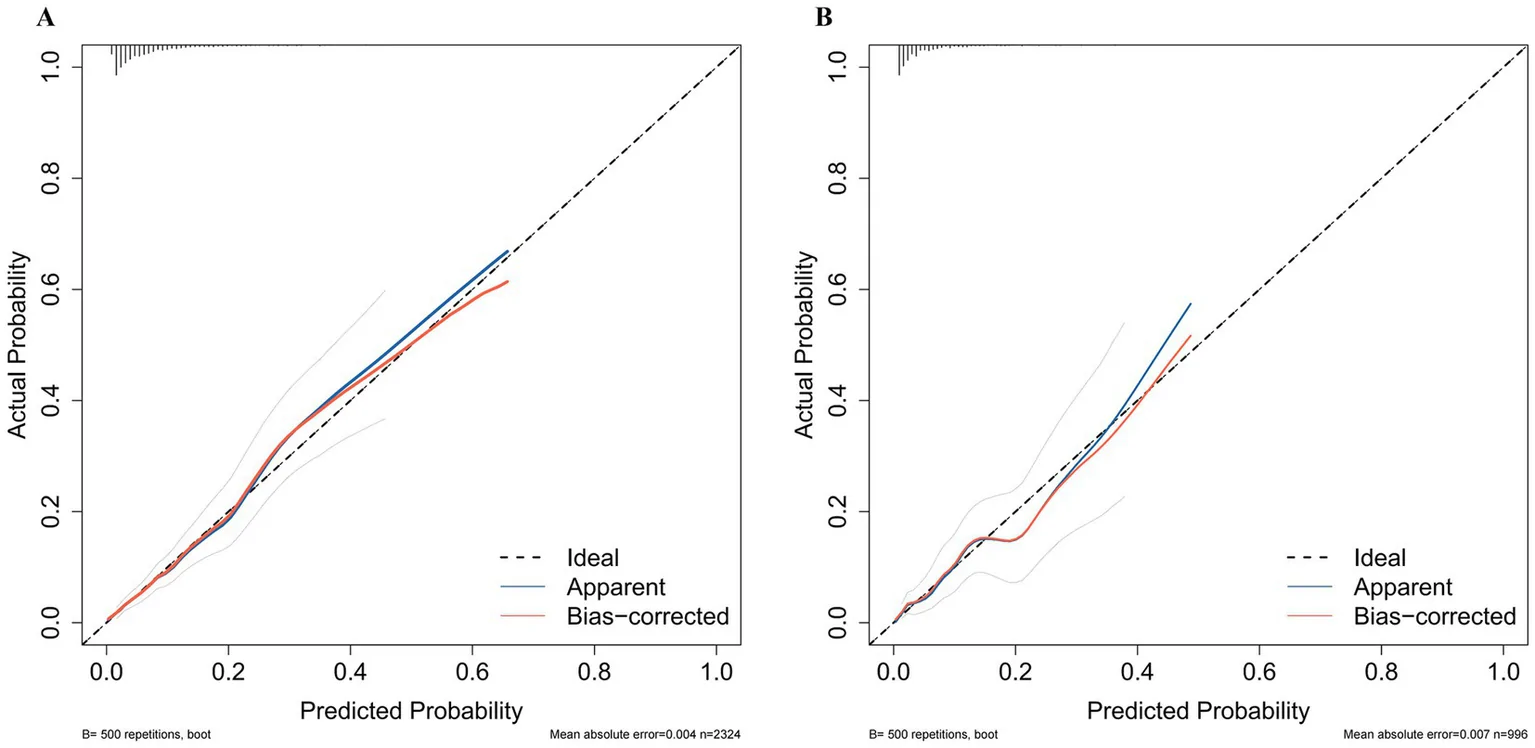
Calibration curves of the prediction model. **(A)** Training set; **(B)** Validation set.

**Figure 5:**
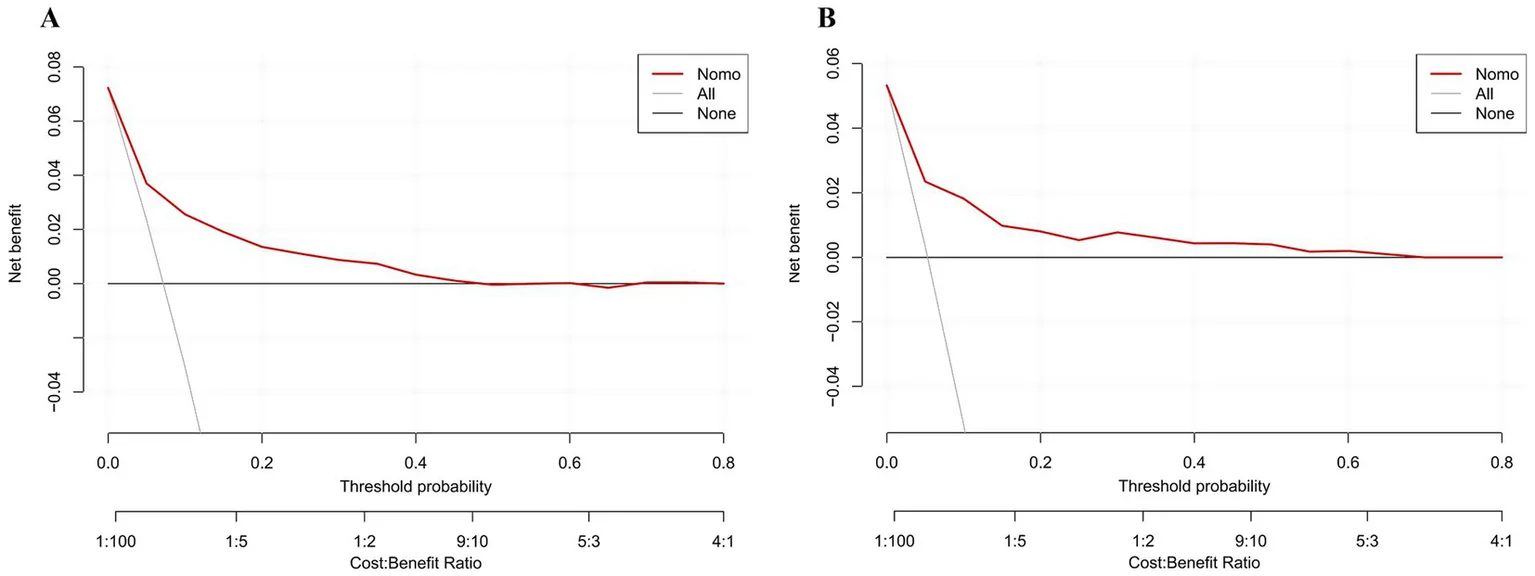
Decision curve analysis of the prediction model. **(A)** Training set; **(B)** Validation set.

### Sensitivity analysis

We divided the 13 cities in the JSNRFCN into three datasets according to different geographic regions: Southern Jiangsu, Central Jiangsu, and Northern Jiangsu. Each of these three datasets was subsequently incorporated into the LR model for sensitivity analysis. This approach addresses the variability in center size across the 30 participating NICUs and provides stable performance estimates across clinically meaningful subgroups. Specifically, the Southern Jiangsu region included 2,123 preterm infants, the Central Jiangsu region included 416 preterm infants, and the Northern Jiangsu region included 781 preterm infants. The results showed that the AUC values for the sensitivity analyses in the Southern Jiangsu, Central Jiangsu, and Northern Jiangsu datasets were 0.770 (95% CI: 0.732–0.808), 0.729 (95% CI: 0.568–0.890), and 0.885 (95% CI: 0.835–0.935), respectively. The Hosmer-Lemeshow test *p*-values were 0.652, 0.052, and 0.314, respectively. The ROC curves and calibration curves for all subgroups are presented in Supplementary Figures 7, 8.

As a sensitivity analysis to assess the robustness of our findings to departures from the MAR assumption, we performed NARFCS-based multiple imputation across a range of δ adjustment parameters (−1.5 to +1.5 in 0.5 increments). The AUC values under the tested scenarios ranged from 0.7770 to 0.7772, with a maximum deviation of 0.008 from the MAR-based AUC (0.785). The coefficient estimates for all six predictors retained their direction and relative magnitude across all scenarios, indicating that the model performance remained stable across plausible deviations from MAR (Supplementary Table 11).

Furthermore, as a sensitivity analysis, we applied SMOTE to address class imbalance. Comparison of baseline characteristics between the SMOTE-augmented training set and the original training set revealed statistically significant differences across all variables, as detailed in Supplementary Table 12. The SMOTE-enhanced model yielded a training set AUC of 0.797 (95% CI: 0.783–0.811) and a validation set AUC of 0.805 (95% CI: 0.740–0.870). Delong’s test comparing the original and SMOTE-enhanced models showed no significant difference (*p* > 0.05), indicating that the LR model was robust to class imbalance. The Brier score for the SMOTE model was 0.180 in the training set and 0.138 in the validation set—both higher than the corresponding scores for the original model (0.058 and 0.043, respectively)—which is expected, as SMOTE alters the outcome distribution and introduces additional uncertainty into probability estimation. Calibration assessment, as shown in Supplementary Figure 9, indicated that model calibration remained within an acceptable range. These findings support the decision to retain the original model, which offers superior calibration performance and accurately reflects the natural disease prevalence—a key requirement for clinical risk estimation.

### Nomogram construction and visualization

Based on the LR analysis results, a prediction model consisting of six predictors was constructed (Table 2). The model is presented as a nomogram. According to the different variables, a vertical line is drawn at the top of the nomogram to obtain the corresponding score. The scores of each variable are summed to obtain the total score, and the corresponding total risk score is obtained at the bottom of the nomogram. For example, if a preterm infant has a gestational age of 30 weeks, a birth weight of approximately 1,500 g, does not undergo endotracheal intubation, chest compressions, or surfactant administration in the DR at birth, and the mother is diagnosed with chorioamnionitis, the total score is 377 points. This score corresponds to a probability of 0.15 on the nomogram, meaning that the probability of SIVH occurrence in this case is 15%, as shown in Figure 6. To facilitate the clinical application of the model, we deployed the model on an online web page, accessible at https://SIVH-by-shenfei.shinyapps.io/New-SIVH/.

**Table 2:** Multivariable logistic regression analysis of SIVH.

Characteristics	*B*	SE	OR (95%*CI*)	*Z*	*P*
(Intercept)	7.12	1.565	1236.574 (57.526–26581.138)	4.549	<0.001
Gestational age	−0.273	0.063	0.761 (0.672–0.862)	−4.309	<0.001
Chorioamnionitis	0.912	0.268	2.489 (1.471–4.210)	3.398	0.001
Birth weight	−0.001	<0.001	0.999 (0.998–1.000)	−2.913	0.004
Chest compressions in the DR	0.71	0.334	2.034 (1.057–3.917)	2.125	0.034
PS administration in the DR	1.296	0.629	3.653 (1.065–12.535)	2.060	0.039
Endotracheal intubation in the DR, n (%)	0.451	0.262	1.569 (0.939–2.623)	1.720	0.085

PS, pulmonary surfactant; SIVH, severe intraventricular hemorrhage; DR, delivery room; Based on the multivariable logistic regression analysis, the full prediction equation is: Logit(P) = 7.120–0.273 × GA + 0.912 × Chorioamnionitis − 0.001 × BW + 0.710 × Chest compressions in the DR + 1.296 × PS administration in the DR + 0.451 × Endotracheal intubation in the DR, where GA represents gestational age (weeks), BW birth weight (grams), and the remaining variables are binary indicators (1 = yes, 0 = no) for chorioamnionitis, chest compressions in the delivery room, surfactant administration in the delivery room, and endotracheal intubation in the delivery room. The corresponding SIVH risk probability is calculated as p = 1/[1 + exp(−Logit(P))].

**Figure 6:**
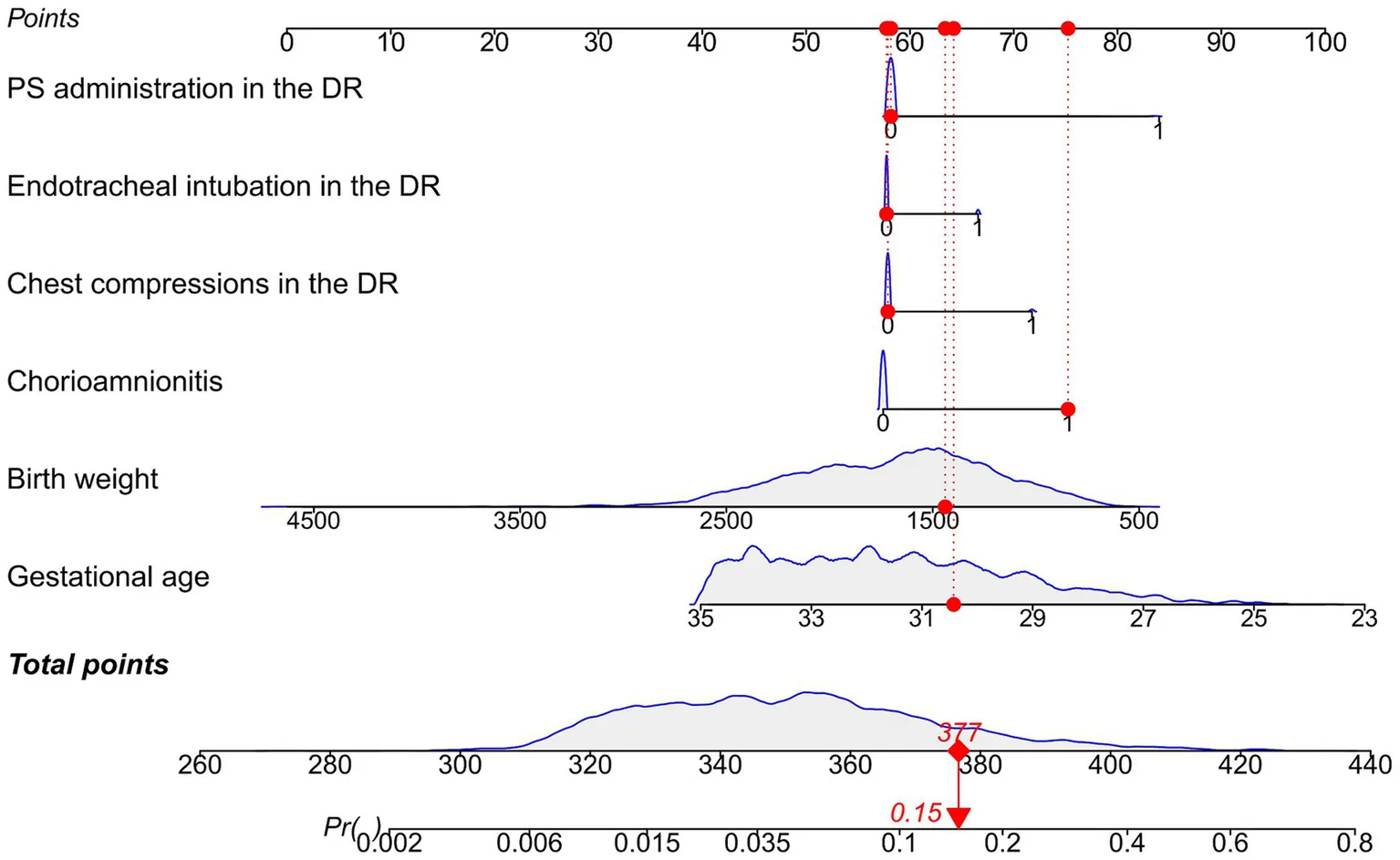
Nomogram for predicting the risk of SIVH.

## Discussion

This study systematically compared the performance of eight ML algorithms in predicting the risk of SIVH in preterm infants based on a large-scale multicenter cohort. We found that the LR model, composed of six perinatal indicators, demonstrated good discrimination in the independent internal validation set, the temporal validation cohort, 1,000 bootstrap resampling tests, and geographic subgroup sensitivity analyses. It also exhibited good calibration and clinical net benefit, making it the optimal model among the eight ML approaches. Our meta-analysis summarized the discrimination of seven previous prediction models for SIVH in preterm infants, with a pooled AUC of 0.805 (95% CI: 0.756–0.853) (8). The model developed in this study, incorporating only prenatal and intrapartum variables, achieved an AUC nearly comparable to that value. This not only enables accurate prediction but also allows for early clinical prediction, while to some extent avoiding bias caused by reverse temporal causality between variables and the SIVH outcome. The sensitivity analysis performed after excluding infants with mild IVH from the temporal validation cohort yielded an AUC of 0.911, indicating that the model maintains excellent discriminative ability even when required to differentiate between two severe grades of hemorrhage.

When comparing the eight ML algorithms, LR model achieved the highest mean cross-validated AUC in the training set and maintained the best discriminative ability in the independent validation set, confirming its generalizability to unseen data. In the temporal validation cohort, LR remained robust, slightly below XGBoost but superior to all other algorithms. Complex models such as RF and KNN did not outperform LR in the training set, instead ranking lower in cross-validated AUC. These observations suggest that the true associations in our dataset may be predominantly driven by a limited number of strong predictors, principally gestational age and birth weight, and that the relationships between these variables and the outcome are largely linear. Under such conditions, the added flexibility of complex algorithms confers no meaningful advantage; these models may capture noise and spurious interactions that fail to generalize to new populations (18, 19). The LR model, with its inherent simplicity and interpretability, effectively avoids overfitting and achieves superior generalizability. These findings reinforce that in clinical prediction modeling, particularly when the predictor space is limited and relationships are relatively straightforward, simpler models are often preferable to more complex alternatives (20). The widespread use of logistic regression in medical research further supports its selection when predictor relationships are predominantly linear and interpretability is paramount (21).

The six predictors identified in this study all have clear clinical and biological plausibility. Low gestational age and low birth weight reflect the degree of immaturity of the germinal matrix vasculature and deficits in cerebral autoregulation in preterm infants. Consistent with the existing literature, these two predictors are often considered the strongest factors for SIVH in preterm infants and are frequently used together in prediction models for SIVH in this population (12, 22).

Maternal chorioamnionitis is the only antenatal infection-related predictor in this model. Duan et al. (23) found that moderate-to-severe chorioamnionitis is closely associated with early-onset sepsis, increased SIVH, and long-term cognitive impairment in very preterm infants. A systematic review and meta-analysis involving over 40,000 infants further confirmed that chorioamnionitis is not only a risk factor for IVH but also a risk factor for increased prematurity severity and clinical instability (24). Ushida et al. (25) constructed multiple prediction models for SIVH in preterm infants based on 12 variables, including similar prenatal and intrapartum factors, achieving AUCs ranging from 0.765 to 0.774, demonstrating the feasibility of this strategy. However, our model incorporates only half the number of predictors used in the model by Ushida et al. (25) yet achieves better predictive performance. Our advantage may lie precisely in the inclusion of DR resuscitation variables. Endotracheal intubation, chest compressions, and surfactant administration in the DR are markers of the intensity of DR resuscitation. Together, these variables reflect the severity of respiratory distress in preterm infants at birth. Both severe respiratory distress and vigorous manipulations can impair the already fragile cerebral autoregulatory function of preterm infants, leading to passive pressure fluctuations in the germinal matrix vasculature and thereby inducing rupture and hemorrhage (26). The inclusion of these variables not only makes the model more parsimonious but also further enhances its predictive performance.

Our model demonstrates potential value in clinical practice. According to statistics, approximately 50% of IVH occurs within the first 24 h after birth in preterm infants, and about 90% occurs within the first 72 h, indicating that there is only a brief time window for hemorrhage prevention and intervention (27). Although some models incorporating postnatal predictors such as routine blood tests and mechanical ventilation parameters may achieve higher predictive performance, including such variables not only introduces time delays but may also lead to reverse causation, thereby undermining the early warning value of the model (28–29). Variables such as gestational age at birth, birth weight, DR resuscitation measures, and maternal chorioamnionitis are readily available. Using an online web tool, clinicians can complete risk stratification immediately after birth, creating the possibility for early implementation of preventive interventions. However, these potential benefits should be viewed as hypotheses requiring prospective validation rather than established effects of model application.

The optimal timing and frequency of the first cranial ultrasound remain uncertain, with published guidelines varying. Vries et al. (27) recommend performing the first ultrasound on the day of admission for all preterm infants with gestational age <32 weeks, whereas guidelines from several pediatric societies, including the Canadian Paediatric Society, often delay screening until 4–7 days after birth (30). Delaying screening may miss the opportunity to identify infants at imminent risk of hemorrhage and to implement neuroprotective measures during the most vulnerable window (31). Therefore, we recommend that future guideline updates incorporate a risk-stratified screening protocol, in which the timing of the first cranial ultrasound is guided by a validated prediction model. However, this strategy requires prospective validation before it can be recommended for routine practice. For infants identified as high-risk by the model, clinicians can justifiably advance the first cranial ultrasound to within 24 h after birth, while still adhering to the principle of individualized rather than routine imaging screening.

Several limitations of this study warrant consideration. First, although derived from a prospectively maintained multicenter cohort, the analysis was retrospective in nature, which may have introduced selection and information bias. Second, cranial ultrasound examinations were performed at varying time points within the first week after birth, and the outcome was defined as the highest IVH grade observed during hospitalization. Because no baseline ultrasound was obtained at birth or at the time of model application, some infants may already have developed SIVH before their first ultrasound. Thus, our model predicts SIVH detected during hospitalization rather than strictly incident hemorrhage occurring after model application. Third, our cohort was drawn from a respiratory support registry, and all included infants received noninvasive or invasive respiratory support. Our findings may therefore also not be applicable to preterm infants who do not require respiratory support. Fourth, chorioamnionitis was diagnosed using clinical criteria rather than placental histopathology, which may have led to misclassification of intrauterine infection severity and potentially underestimated its true association with SIVH. Fifth, surfactant administration in the DR was an infrequent exposure (25 infants, 0.75%), resulting in a relatively wide confidence interval for the corresponding odds ratio (3.653, 95% CI: 1.065–12.535). While LASSO regularization suggested stable coefficient estimates, this finding should be interpreted with caution and warrants confirmation in larger cohorts. Sixth, a substantial number of infants (n = 359) were excluded from the analysis. Compared with included infants, the excluded group had significantly lower gestational age and birth weight, and higher rates of DR resuscitation, confirming that they represented a higher-acuity subgroup. Their exclusion likely led to underestimation of the true SIVH incidence and may limit generalizability to the most critically ill preterm infants. However, inclusion of these infants was not feasible as ultrasound data were unavailable; the comparison provided in Supplementary Table 5 offers context for interpreting this bias. Finally, although we performed temporal validation and geographic subgroup sensitivity analyses, these represent internal-external validation rather than fully independent external validation using data from different populations or healthcare systems. Formal external validation in broader geographic regions, different racial populations, and NICUs with varying levels of care is needed before widespread clinical implementation.

In conclusion, we developed and preliminarily validated a LR model based on six core perinatal variables. This model provides clinicians with a user-friendly tool for SIVH risk stratification in preterm infants who require respiratory support. By identifying high-risk infants, it can guide the implementation of individualized preventive strategies, such as optimizing DR management, enhancing meticulous hemodynamic and respiratory support regulation within the first few hours of life, and increasing the frequency of neuroimaging monitoring. These efforts have the potential to improve outcomes, although this hypothesis requires prospective evaluation. Future multicenter external validation and prospective interventional studies are needed to further assess its clinical value.

## Data Availability

The data that support the findings of this study are not publicly available due to data regulations of JSNRFCN. However, the dataset used during this study is available from the corresponding author upon reasonable request. Requests to access these datasets should be directed to Yang Yang, yy860508@163.com.

## Glossary

IVH: intraventricular hemorrhage
SIVH: severe intraventricular hemorrhage
DR: delivery room
NICUs: neonatal intensive care units
LASSO: least absolute shrinkage and selection operator
ML: machine learning
LR: logistic regression
NN: neural network
GBM: gradient boosting machine
XGBoost: extreme gradient boosting
RF: random forest
SVM: support vector machine
KNN: K-nearest neighbors
Adaboost: adaptive boosting
AUC: area under the curve
ROC: receiver operating characteristic
DCA: decision curve analysis
TRIPOD: Transparent Reporting of a Multivariable Prediction Model for Individual Prognosis or Diagnosis
MICE: multiple imputation by chained equations
MAR: missing at random
MCAR: missing completely at random
VIF: variance inflation factor
IC: information criteria
AIC: akaike information criterion
BIC: bayesian information criterion
NARFCS: not at random fully conditional specification
SMOTE: synthetic minority over-sampling technique
