# Supplementary material for: Development of a machine learning-based multicenter study for predicting severe intraventricular hemorrhage in preterm infants using perinatal variables
Source: Front Neurol. 2026 Oct 1;17:1953566. doi: 10.3389/fneur.2026.1953566 (PMC13668575; doi:10.3389/fneur.2026.1953566)
Supplement: Supplementary file 1 [file Table_1.docx]

**Supplementary table 1. Distribution of missing data across the dataset**

| **Variable** | **Total Entries** | **Missing Values** | **Missing Percentage** |
| --- | --- | --- | --- |
| Antenatal steroids | 3320 | 428 | 12.89% |
| Fetal distress | 3320 | 348 | 10.48% |
| Clear amniotic fluid | 3320 | 50 | 1.51% |
| 5min Apgar score | 3320 | 42 | 1.27% |
| 1min Apgar score | 3320 | 38 | 1.14% |
| Mother age | 3320 | 4 | 0.12% |

**Supplementary table 2. AIC and BIC values for the multiply imputed datasets (training set only)**

| **Data set** | **null deviance** | **df null** | **logLik** | **AIC** | **BIC** | **deviance** | **df residual** | **nobs** |
| --- | --- | --- | --- | --- | --- | --- | --- | --- |
| 1 | 1206 | 2323 | -493 | 1041 | 1202 | 985 | 2296 | 2324 |
| 2 | 1206 | 2323 | -493 | 1042 | 1203 | 986 | 2296 | 2324 |
| 3 | 1206 | 2323 | -493 | 1042 | 1203 | 986 | 2296 | 2324 |
| 4 | 1206 | 2323 | -493 | 1042 | 1203 | 986 | 2296 | 2324 |
| 5 | 1206 | 2323 | -493 | 1043 | 1204 | 987 | 2296 | 2324 |

*Note*: AIC, akaike information criterion; BIC, bayesian information criterion.

**Supplementary table 3. Distribution of sIVH cases among preterm infants by gestational age**

| **Gestational age** | **No. of infants** | **SIVH (%)** |
| --- | --- | --- |
| 23-23^+6^ | 1 | 1 (100.00) |
| 24-24^+6^ | 8 | 6 (75.00) |
| 25-25^+6^ | 24 | 13 (54.17) |
| 26-26^+6^ | 58 | 19 (32.76) |
| 27-27^+6^ | 118 | 32 (27.12) |
| 28-28^+6^ | 197 | 24 (12.18) |
| 29-29^+6^ | 327 | 38 (11.62) |
| 30-30^+6^ | 460 | 31 (6.74) |
| 31-31^+6^ | 545 | 22 (4.04) |
| 32-32^+6^ | 545 | 21 (3.85) |
| 33-33^+6^ | 491 | 7 (1.43) |
| 34-34^+6^ | 546 | 7 (1.28) |

*Note*: SIVH, severe intraventricular hemorrhage.

**Supplementary table 4. Comparison of baseline characteristics between the training and validation sets**

| **Variables** | **Total**  **(n = 3320)** | **Validation set**  **(n = 996)** | **Training set**  **(n = 2324)** | **Statistic** | ***P*** |
| --- | --- | --- | --- | --- | --- |
| **Sex, n (%)** |  |  |  | 1.907 | 0.167 |
| female | 1395 (42) | 437 (44) | 958 (41) |  |  |
| male | 1925 (58) | 559 (56) | 1366 (59) |  |  |
| **Gestational age, Median (Q1,Q3)** | 31.86 (30.14, 33.43) | 31.86 (30.29, 33.43) | 31.71 (30.14, 33.29) | 1199745 | 0.094 |
| **Multiple births, n (%)** |  |  |  | 0.74 | 0.39 |
| no | 2547 (77) | 754 (76) | 1793 (77) |  |  |
| yes | 773 (23) | 242 (24) | 531 (23) |  |  |
| **Birth weight, Median (Q1,Q3)** | 1650 (1350, 2030) | 1690 (1390, 2060) | 1650 (1350, 2030) | 1205565.5 | 0.057 |
| **SGA, n (%)** |  |  |  | 0.112 | 0.738 |
| no | 3099 (93) | 927 (93) | 2172 (93) |  |  |
| yes | 221 (7) | 69 (7) | 152 (7) |  |  |
| **IVF, n (%)** |  |  |  | 0.223 | 0.636 |
| no | 2837 (85) | 856 (86) | 1981 (85) |  |  |
| yes | 483 (15) | 140 (14) | 343 (15) |  |  |
| **Delivery mode, n (%)** |  |  |  | 0.778 | 0.678 |
| vaginal delivery | 1150 (35) | 338 (34) | 812 (35) |  |  |
| elective cesarean section | 1332 (40) | 411 (41) | 921 (40) |  |  |
| emergency cesarean section | 838 (25) | 247 (25) | 591 (25) |  |  |
| **Fetal distress, n (%)** |  |  |  | 0.018 | 0.894 |
| no | 3025 (91) | 906 (91) | 2119 (91) |  |  |
| yes | 295 (9) | 90 (9) | 205 (9) |  |  |
| **Clear amniotic fluid, n (%)** |  |  |  | 0.333 | 0.564 |
| no | 320 (10) | 101 (10) | 219 (9) |  |  |
| yes | 3000 (90) | 895 (90) | 2105 (91) |  |  |
| **Normal amniotic fluid volume, n (%)** |  |  |  | 3.45 | 0.063 |
| no | 261 (8) | 92 (9) | 169 (7) |  |  |
| yes | 3059 (92) | 904 (91) | 2155 (93) |  |  |
| **1min Apgar, Median (Q1,Q3)** | 8 (7, 9) | 8 (7, 9) | 8 (7, 9) | 1194928 | 0.126 |
| **5min Apgar, Median (Q1,Q3)** | 9 (8, 9) | 9 (8, 9) | 9 (8, 9) | 1181954 | 0.312 |
| **No Resuscitation, n (%)** |  |  |  | 3.415 | 0.065 |
| no | 1449 (44) | 410 (41) | 1039 (45) |  |  |
| yes | 1871 (56) | 586 (59) | 1285 (55) |  |  |
| **Endotracheal intubation in the DR, n (%)** |  |  |  | 2.902 | 0.088 |
| no | 3112 (94) | 945 (95) | 2167 (93) |  |  |
| yes | 208 (6) | 51 (5) | 157 (7) |  |  |
| **Chest compressions in the DR, n (%)** |  |  |  | 1.954 | 0.162 |
| no | 3217 (97) | 972 (98) | 2245 (97) |  |  |
| yes | 103 (3) | 24 (2) | 79 (3) |  |  |
| **PS administration in the DR, n (%)** |  |  |  | 0 | 1 |
| no | 3295 (99) | 988 (99) | 2307 (99) |  |  |
| yes | 25 (1) | 8 (1) | 17 (1) |  |  |
| **Antenatal steroids, n (%)** |  |  |  | 0.525 | 0.769 |
| no | 806 (24) | 250 (25) | 556 (24) |  |  |
| incomplete | 1004 (30) | 298 (30) | 706 (30) |  |  |
| complete | 1510 (45) | 448 (45) | 1062 (46) |  |  |
| **Mother age, Median (Q1,Q3)** | 30 (27, 33) | 30 (27, 33) | 30 (27, 33) | 1172053 | 0.56 |
| **GDM, n (%)** |  |  |  | 0.069 | 0.792 |
| no | 2763 (83) | 832 (84) | 1931 (83) |  |  |
| yes | 557 (17) | 164 (16) | 393 (17) |  |  |
| **Placental abruption, n (%)** |  |  |  | 0.017 | 0.897 |
| no | 3174 (96) | 951 (95) | 2223 (96) |  |  |
| yes | 146 (4) | 45 (5) | 101 (4) |  |  |
| **Gestationa hypertension, n (%)** |  |  |  | 0.365 | 0.546 |
| no | 2799 (84) | 846 (85) | 1953 (84) |  |  |
| yes | 521 (16) | 150 (15) | 371 (16) |  |  |
| **Chronic hypertension, n (%)** |  |  |  | 0.063 | 0.801 |
| no | 3258 (98) | 976 (98) | 2282 (98) |  |  |
| yes | 62 (2) | 20 (2) | 42 (2) |  |  |
| **Maternal heart diseases, n (%)** |  |  |  | 0.363 | 0.547 |
| no | 3301 (99) | 992 (100) | 2309 (99) |  |  |
| yes | 19 (1) | 4 (0) | 15 (1) |  |  |
| **Maternal severe anaemia, n (%)** |  |  |  | 1.505 | 0.22 |
| no | 3283 (99) | 981 (98) | 2302 (99) |  |  |
| yes | 37 (1) | 15 (2) | 22 (1) |  |  |
| **Chorioamnionitis, n (%)** |  |  |  | 0.558 | 0.455 |
| no | 3154 (95) | 951 (95) | 2203 (95) |  |  |
| yes | 166 (5) | 45 (5) | 121 (5) |  |  |

*Note*: IVF, in vitro fertilization; SGA, small for gestational age; PS, pulmonary surfactant; SIVH, severe intraventricular hemorrhage; GDM, gestational diabetes mellitus; DR, delivery room.

**Supplementary Table 5. Comparison of baseline characteristics between included and excluded infants (raw, unimputed data)**

| **Variables** | **Total**  **(n = 3679)** | **Excluded infants**  **(n = 359)** | **Included infants**  **(n = 3320)** | **Statistic** | ***P*** |
| --- | --- | --- | --- | --- | --- |
| **Sex, n (%)** |  |  |  | 0.054 | 0.816 |
| female | 1543 (42) | 148 (41) | 1395 (42) |  |  |
| male | 2136 (58) | 211 (59) | 1925 (58) |  |  |
| **Gestational age, Median (Q1,Q3)** | 31.57 (29.86, 33.29) | 29.71 (28, 31.43) | 31.86 (30.14, 33.43) | -12.65106 | < 0.001 |
| **Multiple births, n (%)** |  |  |  | 0 | 0.997 |
| no | 2823 (77) | 276 (77) | 2547 (77) |  |  |
| yes | 856 (23) | 83 (23) | 773 (23) |  |  |
| **Birth weight, Median (Q1,Q3)** | 1620 (1300, 2005) | 1250 (1004, 1667.5) | 1650 (1350, 2030) | -12.37659 | < 0.001 |
| **SGA, n (%)** |  |  |  | 15.824 | < 0.001 |
| no | 3413 (93) | 314 (87) | 3099 (93) |  |  |
| yes | 266 (7) | 45 (13) | 221 (7) |  |  |
| **IVF, n (%)** |  |  |  | 0.03 | 0.863 |
| no | 3142 (85) | 305 (85) | 2837 (85) |  |  |
| yes | 537 (15) | 54 (15) | 483 (15) |  |  |
| **Delivery mode, n (%)** |  |  |  | 18.527 | < 0.001 |
| vaginal delivery | 1313 (36) | 163 (45) | 1150 (35) |  |  |
| elective cesarean section | 1441 (39) | 109 (30) | 1332 (40) |  |  |
| emergency cesarean section | 925 (25) | 87 (24) | 838 (25) |  |  |
| **Fetal distress, n (%)** |  |  |  | 3.083 | 0.079 |
| no | 2985 (91) | 277 (88) | 2708 (91) |  |  |
| yes | 302 (9) | 38 (12) | 264 (9) |  |  |
| **Clear amniotic fluid, n (%)** |  |  |  | 7.627 | 0.006 |
| no | 364 (10) | 51 (14) | 313 (10) |  |  |
| yes | 3261 (90) | 304 (86) | 2957 (90) |  |  |
| **Normal amniotic fluid volume, n (%)** |  |  |  | 2.864 | 0.091 |
| no | 299 (8) | 38 (11) | 261 (8) |  |  |
| yes | 3380 (92) | 321 (89) | 3059 (92) |  |  |
| **1min Apgar, Median (Q1,Q3)** | 8 (7, 9) | 7 (4, 8) | 8 (7, 9) | -10.54675 | < 0.001 |
| **5min Apgar, Median (Q1,Q3)** | 9 (8, 9) | 8 (7, 9) | 9 (8, 9) | -8.703697 | < 0.001 |
| **No Resuscitation, n (%)** |  |  |  | 33.855 | < 0.001 |
| no | 1664 (45) | 215 (60) | 1449 (44) |  |  |
| yes | 2015 (55) | 144 (40) | 1871 (56) |  |  |
| **Endotracheal intubation in the DR, n (%)** |  |  |  | 109.422 | < 0.001 |
| no | 3392 (92) | 280 (78) | 3112 (94) |  |  |
| yes | 287 (8) | 79 (22) | 208 (6) |  |  |
| **Chest compressions in the DR, n (%)** |  |  |  | 60.99 | < 0.001 |
| no | 3534 (96) | 317 (88) | 3217 (97) |  |  |
| yes | 145 (4) | 42 (12) | 103 (3) |  |  |
| **PS administration in the DR, n (%)** |  |  |  | Fisher | 0.208 |
| no | 3649 (99) | 354 (99) | 3295 (99) |  |  |
| yes | 30 (1) | 5 (1) | 25 (1) |  |  |
| **Antenatal steroids, n (%)** |  |  |  | 3.026 | 0.22 |
| no | 768 (24) | 79 (26) | 689 (24) |  |  |
| incomplete | 980 (31) | 99 (33) | 881 (30) |  |  |
| complete | 1443 (45) | 121 (40) | 1322 (46) |  |  |
| **Mother age, Median (Q1,Q3)** | 30 (27, 33) | 30 (27, 33) | 30 (27, 33) | -1.713907 | 0.11 |
| **GDM, n (%)** |  |  |  | 0.008 | 0.928 |
| no | 3063 (83) | 300 (84) | 2763 (83) |  |  |
| yes | 616 (17) | 59 (16) | 557 (17) |  |  |
| **Placental abruption, n (%)** |  |  |  | 0.026 | 0.873 |
| no | 3516 (96) | 342 (95) | 3174 (96) |  |  |
| yes | 163 (4) | 17 (5) | 146 (4) |  |  |
| **Gestationa hypertension, n (%)** |  |  |  | 0.012 | 0.914 |
| no | 3103 (84) | 304 (85) | 2799 (84) |  |  |
| yes | 576 (16) | 55 (15) | 521 (16) |  |  |
| **Chronic hypertension, n (%)** |  |  |  | 0.003 | 0.955 |
| no | 3611 (98) | 353 (98) | 3258 (98) |  |  |
| yes | 68 (2) | 6 (2) | 62 (2) |  |  |
| **Maternal heart diseases, n (%)** |  |  |  | Fisher | 0.274 |
| no | 3656 (99) | 355 (99) | 3301 (99) |  |  |
| yes | 23 (1) | 4 (1) | 19 (1) |  |  |
| **Maternal severe anaemia, n (%)** |  |  |  | Fisher | 0.305 |
| no | 3636 (99) | 353 (98) | 3283 (99) |  |  |
| yes | 43 (1) | 6 (2) | 37 (1) |  |  |
| **Chorioamnionitis, n (%)** |  |  |  | 0.308 | 0.579 |
| no | 3498 (95) | 344 (96) | 3154 (95) |  |  |
| yes | 181 (5) | 15 (4) | 166 (5) |  |  |

*Note:* Values are presented as median (IQR) for continuous variables and n (%) for categorical variables. Excluded infants comprised 189 who did not undergo ultrasound because of palliative care or death, 119 transferred before ultrasound examination, and 51 with unavailable ultrasound results. Comparisons were performed using the Mann‑Whitney *U* test for continuous variables and the chi‑square test or Fisher's exact test for categorical variables, as appropriate. Raw, unimputed data were used for both groups. IVF, in vitro fertilization; SGA, small for gestational age; PS, pulmonary surfactant; SIVH, severe intraventricular hemorrhage; GDM, gestational diabetes mellitus.

**Supplementary table 6. Baseline characteristics of preterm infants and their mothers in the temporal validation cohort**

| **Variables** | **Total**  **(n = 233)** | **Non-SIVH**  **(n = 184)** | **SIVH**  **(n = 49)** | **statistic** | ***P*** |
| --- | --- | --- | --- | --- | --- |
| **PS administration in the DR, n (%)** |  |  |  | 0.466 | 0.495 |
| no | 204 (88) | 163 (89) | 41 (84) |  |  |
| yes | 29 (12) | 21 (11) | 8 (16) |  |  |
| **Gestational age, Median (Q1,Q3)** | 29.22 ± 2.38 | 29.78 ± 2.15 | 27.09 ± 1.98 | 8.278 | < 0.001 |
| **Endotracheal intubation in the DR, n (%)** |  |  |  | 23.972 | < 0.001 |
| no | 178 (76) | 154 (84) | 24 (49) |  |  |
| yes | 55 (24) | 30 (16) | 25 (51) |  |  |
| **Chest compressions in the DR, n (%)** |  |  |  | Fisher | 0.013 |
| no | 222 (95) | 179 (97) | 43 (88) |  |  |
| yes | 11 (5) | 5 (3) | 6 (12) |  |  |
| **Birth weight, Median (Q1,Q3)** | 1125 (940, 1330) | 1165 (997.5, 1360) | 998 (770, 1100) | 6589 | < 0.001 |
| **Chorioamnionitis, n (%)** |  |  |  | Fisher | 0.013 |
| no | 222 (95) | 179 (97) | 43 (88) |  |  |
| yes | 11 (5) | 5 (3) | 6 (12) |  |  |

*Note*: PS, pulmonary surfactant; SIVH, severe intraventricular hemorrhage; DR, delivery room.

**Supplementary Table 7. Distribution of infrequent delivery room exposures and SIVH events in the training and validation sets.**

| **Variable** | **Overall (n=3,320)** | **Training Set (n=2,324)** | **Validation Set (n=996)** |
| --- | --- | --- | --- |
| PS in DR, n (%) | 25 (0.75) | 17 (0.73) | 8 (0.80) |
| — of which SIVH, n (%) | 6 (24.00) | 5 (29.41) | 1 (12.50) |
| Chest compressions in DR, n (%) | 103 (3.10) | 79 (3.40) | 24 (2.41) |
| — of which SIVH, n (%) | 23 (22.33) | 19 (24.05) | 4 (16.67) |

*Note:* PS, pulmonary surfactant; SIVH, severe intraventricular hemorrhage; DR, delivery room.

**Supplementary Table 8. Complete hyperparameter search grids for all eight algorithms.**

| **Algorithm** | **Parameters** | **Search Range** | **Combinations** |
| --- | --- | --- | --- |
| Logistic Regression | None (default) | N/A | 1 |
| SVM (Radial Basis) | sigma | 0.1, 0.01, 0.001 | 6 |
|  | C | 0.1, 0.5 |  |
| GBM | n.trees | 100 | 4 |
|  | interaction.depth | 2, 3 |  |
|  | shrinkage | 0.1, 0.01 |  |
|  | n.minobsinnode | 5 |  |
| Neural Network | size (hidden units) | 3, 4, 5 | 3 |
|  | decay | 0.6 |  |
| Random Forest | mtry | 2, 3, 4, 5 | 4 |
| Xgboost | nrounds | 10 | 9 |
|  | max_depth | 3, 4, 5 |  |
|  | eta | 0.1, 0.01, 0.001 |  |
|  | gamma | 0.5 |  |
|  | colsample_bytree | 0.5 |  |
|  | min_child_weight | 1 |  |
|  | subsample | 0.6 |  |
| KNN | kmax | 3, 5, 7, 9, 11, 13, 15 | 7 |
|  | distance | 1 |  |
|  | kernel | optimal |  |
| AdaBoost | mfinal | 30, 50, 100 | 12 |
|  | maxdepth | 2, 3, 4, 5 |  |
|  | coeflearn | Zhu |  |

*Note:* SVM: support vector machine; GBM: gradient boosting machine; Xgboost: extreme gradient boosting; KNN: K-nearest neighbor; Adaboost: adaptive boosting.

**Supplementary Table 9. Final optimal parameters for all eight algorithms.**

| **Algorithm** | **Final Parameters** |
| --- | --- |
| Logistic Regression | None (default) |
| SVM | sigma = 0.001, C = 0.1 |
| GBM | n.trees = 100, interaction.depth = 2, shrinkage = 0.1, n.minobsinnode = 5 |
| Neural Network | size = 5, decay = 0.6 |
| Random Forest | mtry = 2 |
| Xgboost | nrounds = 10, max_depth = 3, eta = 0.1, gamma = 0.5, colsample_bytree = 0.5, min_child_weight = 1, subsample = 0.6 |
| KNN | kmax = 15, distance = 1, kernel = optimal |
| AdaBoost | mfinal = 100, maxdepth = 2, coeflearn = Zhu |

*Note:* SVM: support vector machine; GBM: gradient boosting machine; Xgboost: extreme gradient boosting; KNN: K-nearest neighbor; Adaboost: adaptive boosting.

**Supplementary Table 10. The training set AUC for each algorithm after hyperparameter optimization.**

| **Rank** | **Model** | **Mean AUC** | **SD AUC** |
| --- | --- | --- | --- |
| 1 | Logistic Regression | 0.785 | 0.055 |
| 2 | GBM | 0.784 | 0.050 |
| 3 | Random Forest | 0.769 | 0.066 |
| 4 | Xgboost | 0.767 | 0.034 |
| 5 | SVM | 0.764 | 0.068 |
| 6 | AdaBoost | 0.740 | 0.056 |
| 7 | KNN | 0.699 | 0.073 |
| 8 | Neural Network | 0.671 | 0.101 |

*Note:* SVM: support vector machine; GBM: gradient boosting machine; Xgboost: extreme gradient boosting; KNN: K-nearest neighbor; Adaboost: adaptive boosting; SD: standard deviation; AUC: area under the curve.

**Supplementary Table 11. NARFCS sensitivity analysis: AUC and coefficient stability across δ adjustment parameters.**

| **δ** | **Mean AUC** | **SD AUC** | **Difference from MAR (0.785)** |
| --- | --- | --- | --- |
| -1.5 | 0.7770 | 0.0030 | -0.0080 |
| -1.0 | 0.7770 | 0.0031 | -0.0080 |
| -0.5 | 0.7771 | 0.0032 | -0.0079 |
| 0 | 0.7772 | 0.0031 | -0.0078 |
| +0.5 | 0.7771 | 0.0031 | -0.0079 |
| +1.0 | 0.7771 | 0.0031 | -0.0079 |
| +1.5 | 0.7771 | 0.0031 | -0.0079 |

*Note:* SD: standard deviation; AUC: area under the curve; MAR: missing at random; NARFCS: not at random fully conditional specification.

**Supplementary Table 12. Comparison of baseline characteristics between the SMOTE‑augmented training set and the original training set**

| **Variables** | **SMOTE-Enhanced Model**  **(n = 3836)** | **Original Model**  **(n = 2324)** | **Statistic** | ***P*** |
| --- | --- | --- | --- | --- |
| **SIVH, n (%)** |  |  | 919.741 | < 0.001 |
| no | 2156 (56) | 2156 (93) |  |  |
| yes | 1680 (44) | 168 (7) |  |  |
| **Gestational age, Median (Q1,Q3)** | 30.83 (29, 32.57) | 31.71 (30.14, 33.29) | -13.511 | < 0.001 |
| **Birth weight, Median (Q1,Q3)** | 1490 (1151.59, 1860) | 1650 (1350, 2030) | -12.836 | < 0.001 |
| **Endotracheal intubation in the DR, n (%)** |  |  | 44.567 | < 0.001 |
| no | 3373 (88) | 2167 (93) |  |  |
| yes | 463 (12) | 157 (7) |  |  |
| **Chest compressions in the DR, n (%)** |  |  | 27.215 | < 0.001 |
| no | 3586 (93) | 2245 (97) |  |  |
| yes | 250 (7) | 79 (3) |  |  |
| **PS administration in the DR, n (%)** |  |  | 8.263 | 0.004 |
| no | 3774 (98) | 2307 (99) |  |  |
| yes | 62 (2) | 17 (1) |  |  |
| **Chorioamnionitis, n (%)** |  |  | 22.817 | < 0.001 |
| no | 3510 (92) | 2203 (95) |  |  |
| yes | 326 (8) | 121 (5) |  |  |

*Note:* PS, pulmonary surfactant; SIVH, severe intraventricular hemorrhage; DR, delivery room; SMOTE: synthetic minority over-sampling technique.
