# Supplementary material for: Development of a machine learning-based multicenter study for predicting severe intraventricular hemorrhage in preterm infants using perinatal variables
Source: Front Neurol. 2026 Oct 1;17:1953566. doi: 10.3389/fneur.2026.1953566 (PMC13668575; doi:10.3389/fneur.2026.1953566)
Supplement: Supplementary file 2 [file Table_2.docx]

Supplementary Figure 1. Performance comparison of the eight machine learning models in the validation cohort, including F1 score, precision, specificity, sensitivity, and accuracy

Supplementary Figure 2. Calibration curves of the eight machine learning models in the internal validation set (A) and the temporal validation cohort (B)

Supplementary Figure 3. Decision curve analysis (DCA) of the eight machine learning models in the internal validation set (A) and the temporal validation cohort (B)

Supplementary Figure 4. Performance metrics (F1 score, precision, specificity, sensitivity, and accuracy) of the eight machine learning models in the temporal validation cohort

Supplementary Figure 5. Temporal validation of the logistic regression model for distinguishing grade IV from grade III IVH. (A) ROC curve; (B) Calibration curve; (C) DCA curve

Supplementary Figure 6. Receiver operating characteristic (ROC) curve of the logistic regression model after 1,000 bootstrap resampling iterations for internal validation in the training set

Supplementary Figure 7. ROC curves for sensitivity analysis across three regions. Test AUC1: Southern Jiangsu; Test AUC2: Central Jiangsu; Test AUC3: Northern Jiangsu

Supplementary Figure 8. Calibration curves for sensitivity analysis across three regions. (A) Southern Jiangsu; (B) Central Jiangsu; (C) Northern Jiangsu

**Supplementary Figure 9. The calibration of the SMOTE model. (A) Training set; (B) Validation set.**
